# Autocrine Regulation of UVA-Induced IL-6 Production via Release of ATP and Activation of P2Y Receptors

**DOI:** 10.1371/journal.pone.0127919

**Published:** 2015-06-01

**Authors:** Ayumi Kawano, Remi Kadomatsu, Miyu Ono, Shuji Kojima, Mitsutoshi Tsukimoto, Hikaru Sakamoto

**Affiliations:** 1 Radioisotope Research Laboratory, School of Pharmacy, Kitasato University, Shirokane, Minato-ku Tokyo, Japan; 2 Department of Radiation Biosciences, Faculty of Pharmaceutical Sciences, Tokyo University of Science, Yamazaki, Noda-shi Chiba, Japan; Albert Einstein College of Medicine, UNITED STATES

## Abstract

Extracellular nucleotides, such as ATP, are released from cells in response to various stimuli and act as intercellular signaling molecules through activation of P2 receptors. Exposure to the ultraviolet radiation A (UVA) component of sunlight causes molecular and cellular damage, and in this study, we investigated the involvement of extracellular nucleotides and P2 receptors in the UVA-induced cellular response. Human keratinocyte-derived HaCaT cells were irradiated with a single dose of UVA (2.5 J/cm^2^), and ATP release and interleukin (IL)-6 production were measured. ATP was released from cells in response to UVA irradiation, and the release was blocked by pretreatment with inhibitors of gap junction hemichannels or P2X7 receptor antagonist. IL-6 production was increased after UVA irradiation, and this increase was inhibited by ecto-nucleotidase or by antagonists of P2Y11 or P2Y13 receptor. These results suggest that UVA-induced IL-6 production is mediated by release of ATP through hemichannels and P2X7 receptor, followed by activation of P2Y11 and P2Y13 receptors. Interestingly, P2Y11 and P2Y13 were associated with the same pattern of IL-6 production, though they trigger different intracellular signaling cascades: Ca^2+^-dependent and PI3K-dependent, respectively. Thus, IL-6 production in response to UVA-induced ATP release involves at least two distinct pathways, mediated by activation of P2Y11 and P2Y13 receptors.

## Introduction

Long-term exposure to sunlight causes various detrimental effects to the skin, including skin cancer and premature aging (photoaging), characterized by epidermal hyperplasia, wrinkles and mottled pigmentation [1, 2]. Solar radiation at the earth’s surface includes short-wavelength ultraviolet (UVB, 280–320 nm) and long-wavelength ultraviolet (UVA, 320–400 nm) components. Although both are injurious, UVA radiation has been considered less carcinogenic and mutagenic because of its limited ability to cause direct DNA damage [3]. For this reason, fewer studies have been performed to examine the molecular and immunological changes induced by UVA in the skin. However, UVA accounts for about 95% of the total UV irradiation [4] and can penetrate the epidermis and reach the basal dermal layer [5, 6]. UVA irradiation causes major changes in skin connective tissues as a result of the degradation of structural components of the extracellular matrix, in both epidermis and dermis [7, 8]. Since it has been suggested that alteration of skin tissues induces cutaneous aging, it is possible that UVA exposure also plays an important role in the development of photoaging.

The epidermis consists mainly of keratinocytes. Cytokines released from keratinocytes influence the migration of inflammatory cells, have possible systemic effects on the immune system, influence keratinocyte proliferation and differentiation, and also affect the production of other cytokines by keratinocytes [9]. UV irradiation strongly promotes the expression in keratinocytes of proinflammatory cytokines, such as interleukin (IL)-1β, IL-6 and tumor necrosis factor (TNF)-α [10, 11]. Induction of inflammatory cytokines by UV, leading to overall skin inflammation, is significant contributor in the photoaging process in skin. The levels of matrix metalloproteinases (MMPs), which are involved in proteolysis of extracellular matrix proteins, are elevated in UV-irradiated skin long before the appearance of visible photoaging [12, 13]. IL-6 released from UV-irradiated-keratinocytes can induce MMP-1 production in fibroblasts [14], and IL-6-neutralizing antibodies are able to block UV-induced MMP-1 production in fibroblasts [15]. These findings suggest that IL-6 might contribute substantially to the progression of photoaging.

Adenosine 5’-triphosphate (ATP) is a key messenger molecule for cell-cell communication [16]. Cytosolic ATP is released into the extracellular space in response to various stimuli, such as shear stress, stretching, hypoxia, inflammation, osmotic swelling and cell death [17]. There are multiple mechanisms of extracellular ATP release, depending upon cell type, including gap junction hemichannels, volume-regulated anion channel (VRAC), ATP-binding cassette (ABC) transporter, and purinergic P2X7 receptor [18]. Extracellular ATP binds specifically to purinergic P2 receptors [16]. P2 receptors have classified into two major groups; ligand-gated ion channel P2X receptors and metabotropic G protein-coupled P2Y receptors [19]. P2Y1, 2, 4 and 6 receptor subtypes are linked to G_q/11_ protein, while P2Y12, 13 and 14 receptors are coupled to G_i_ protein. P2Y11 receptor is coupled to both G_q/11_ and G_s_ protein. In keratinocytes, different purinergic receptors have critical roles in deciding the fate of the cells through regulation of proliferation, differentiation and cell death [20]. Since extracellular ATP stimulates expression and release of IL-6 via P2Y receptors in keratinocytes [21, 22], P2Y receptor signaling is also thought to have an important role in inflammation.

So far, the mechanisms of UVA-induced cellular response remain poorly understood. We have shown that γ- or UVB-irradiation induces ATP release from cells and activation of P2Y receptor in a human keratinocyte cell line, HaCaT [23, 24]. Therefore, in the present study we investigated the mechanism of inflammatory signaling in response to UVA irradiation, focusing on the involvement of purinergic receptors in IL-6 production. Here, we show that UVA exposure induces ATP release, and that the released ATP stimulates IL-6 production through activation of P2Y11 and Y13 receptors.

## Materials and Methods

### Reagents and antibodies

Probenecid and polyoxyethylene sorbitan monolaurate (equivalent to Tween-20) were purchased from Wako Pure Chemical Industries (Osaka, Japan). 5-BDBD, AZ11645373, MRS2179, MRS2578, NF157 and MRS2211 were from Tocris Bioscience (Bristol, UK). BAPTA-AM solution was purchased from Dojindo (Kumamoto, Japan). SQ22536 was purchased from Merck (Darmstadt, Germany). Phospho-Akt rabbit monoclonal antibody (#4060), Akt rabbit monoclonal antibody (#4691), goat horseradish peroxidase (HRP)-conjugated anti-rabbit IgG antibody (#7074) and goat HRP-conjugated anti-mouse IgG antibody (#7076) were purchased from Cell Signaling Technology, Inc. (Beverly, MA). Anti-P2X7 receptor antibody (APR-004), anti-P2Y11 receptor antibody (APR-015) and anti-P2Y13 receptor antibody (APR-017) were purchased from Alomone Labs (Jerusalem, Israel). Mouse anti-β-actin antibody (sc-47778) and goat anti-rabbit IgG-FITC (sc-2012) were purchased from Santa Cruz Biotechnology, Inc. (Santa Cruz, CA). Anti-connexin 43 antibody (ab11370) was purchased from Abcam (Cambridge, UK). Panx1 polyclonal antibody (12595-1-AP) was purchased from Proteintech, Inc (Chicago, IL). Unless otherwise stated, all other reagents were obtained from Sigma-Aldrich (St Louis, MO). All other chemicals used were of the highest purity available.

### Cell culture and irradiation

Immortalized human-derived keratinocytes, HaCaT cells were obtained from American Type Culture Collection (ATCC, Manassas, VA, USA). Cells were cultivated in Dulbecco’s modified Eagle’s medium (low glucose) (Wako), containing 10% fetal bovine serum (HyClone Laboratories, South Logan, UT), 100 units/ml penicillin, and 100 mg/ml streptomycin (Sigma-Aldrich) in an atmosphere of 5% CO_2_, 95% air at 37°C.

Before UVA irradiation, the medium was replaced with Hanks’ based salt solution (HBSS: 136.9 mM NaCl, 5.5 mM KCl, 0.34 mM Na_2_HPO_4_, 0.44 mM KH_2_PO_4_, 0.81 mM MgSO_4_, 1.25 mM CaCl_2_, 5.5 mM D-glucose, 4.2 mM NaHCO_3_ and 10 mM HEPES-NaOH; pH 7.4). The UVA irradiation source was a black light (UVA) lamp (Sankyo Denki, Tokyo, Japan) with an energy peak at 360 nm. The emitted dose was determined by using a radiometer (UVX-36; UVP, Inc., San Gabriel, CA). No UVB was detected when the irradiance was monitored with a UVX-31 sensor (UVP, Inc.).

### Analysis of cell viability

Cell viability was determined by means of 3-(4, 5-dimethylthiazol-2-yl)-5-(3-carboxymethoxyphenyl)-2-(4-sulfophenyl)-2H-tetrazolium (MTS) assay (CellTiter 96 AQueous One Solution Cell Proliferation Assay; Promega, Madison, WI). The CellTiter 96 AQueous One Solution Reagent was directly added to the culture medium according to the manufacturer’s instructions. After incubation at 37°C for 2 h in a humidified air atmosphere containing 5% CO_2_, the absorption at 490 nm was measured with an iMark microplate reader (Bio-Rad, Hercules, CA).

### Reverse Transcription PCR

Total RNA was extracted from cells with ISOGEN reagent (Nippon Gene Co. Ltd., Tokyo, Japan). The first-strand cDNA was synthesized from total RNA with PrimeScript Reverse Transcriptase (Takara Bio, Shiga, Japan). GAPDH mRNA was determined as a positive control. PCR was done by incubating cDNA with appropriate primers (0.5 μM each), Blend Taq polymerase (1.25 U: Toyobo, Osaka, Japan) and deoxynucleotide mix (0.2 mM each: Toyobo). The reaction conditions were as follows: 94°C for 2 min, followed by 30 cycles of 94°C for 30 sec, 56°C for 1 min, and 72°C for 2 min, with final extension at 72°C for 10 min. The primer sequences were as follows: IL-6 (236 bp), 5’-agagtagtgaggaacaagcc-3’ (sense) and 5’-tacatttgccgaagagccct-3’ (anti-sense); GAPDH (258 bp), 5’-agaaggctggggctcatttg-3’ (sense) and 5’-aggggccatccacagtcttc-3’ (anti-sense). The products were then subjected to 2% agarose gel electrophoresis. Bands were stained with ethidium bromide (Wako) and photographed.

### Determination of IL-6 production

Culture supernatant was harvested, and IL-6 was measured by means of ELISA. A 96-well plate was coated with purified anti-human IL-6 mAb (1:500) (eBioscience, San Diego, CA) and incubated overnight at 4°C. The wells were washed with PBS containing 0.05% Tween-20, and nonspecific binding was blocked with PBS containing 1% bovine serum albumin for 1 h at room temperature. The plate was washed, culture supernatant was added, and incubation was continued for 2 h at room temperature. The plate was washed again, and anti-human biotin-conjugated IL-6 mAb (1:500) (eBioscience) was added for 1 h at room temperature. The plate was further washed, and avidin-horseradish peroxidase (Sigma-Aldrich) was added. The plate was incubated for 30 min at room temperature, then washed, and 3,3’,5,5’-tetramethylbenzidine (Sigma-Aldrich) was added. The reaction was stopped by adding 5 N H_2_SO_4_, and the absorption at 450 nm was measured with an iMark microplate reader (Bio-Rad). A standard curve was established with recombinant human IL-6 (eBioscience), and the concentration of IL-6 was estimated by interpolation.

### Measurement of extracellular ATP

Extracellular ATP concentration was measured by using ENLITEN rLuciferase/Luciferin Reagent (Promega). After irradiation, culture supernatant was collected at the indicated time points. Each sample was centrifuged at 600 g for 1 min and 10 μL of the supernatant was used for ATP determination. Luciferin-luciferase reagent (100 μL) was added to the supernatant, and the chemiluminescence was measured with a SpectraMax M5 (Molecular Devices, Orleans, CA). The ATP concentration in each sample was determined by comparing the luminescence of samples with those of standards in the range of 10^–6^–10^–9^ M. For the study on inhibitory effects of various inhibitors on the UVA-induced ATP release, cells were pretreated with GdCl_3_ (50 μM), glibenclamide (100 μM), bafilomycin A (50 nM), CBX (20 μM), AZ11645373 (10 μM), FFA (50 μM), LaCl_3_ (200 μM), probenecid (500 μM), or trovafloxacin (30 μM) for 30 min. At 1 min after irradiation, the supernatants were collected and the ATP content was measured according to the procedures as mentioned above.

### Immunofluorescence staining

Cells were fixed in 3% paraformaldehyde in PBS for 15 min at room temperature and permeabilized in 0.25% Triton X-100 for 10 min. After incubation in blocking buffer (1.5% BSA in PBS) for 1 h, the fixed cells were incubated with primary antibody (connexin 43 1:1000, panx1 1:25) for 24 h at 4°C and with secondary antibody (1:400) for 30 min. Counterstaining with Hoechst 33342 (1 μg/mL) (Dojindo, Kumamoto, Japan) was used to verify the location and integrity of nuclei. Fluorescence images were acquired on a fluorescence microscope (Olympus, Tokyo, Japan) using Olympus DP2-BSW application software.

### Immunoblotting

Cells were washed with cold PBS twice and lysed in cold lysis buffer (10 mM HEPES-NaOH pH 7.4, 1% Triton X-100, 5 mM 2Na-EDTA, 30 mM sodium pyrophosphate, 50 mM sodium fluoride, 1 mM sodium o-vanadate) containing protease inhibitor cocktail (Sigma-Aldrich). Proteins were dissolved in 2 x sample buffer (25% glycerin, 1% SDS, 62.5 mM Tris-Cl, 10 mM DTT) and boiled for 10 min. Aliquots of samples (5 μg per lane) were separated by means of 10% SDS-PAGE and transferred onto a PVDF membrane. Blots were incubated for 2 h at room temperature in Tris-buffered saline containing 0.1% Tween 20 (TBS-T) (10 mM Tris-HCl, 100 mM NaCl, 0.1% Tween 20; pH 7.5) with 1% BSA and incubated overnight at 4°C with primary antibody (phospho-Akt 1:2000, Akt 1:1000, connexin 43 1:5000, panx1 1:500), or incubated for 90 min at room temperature with primary antibody (P2X7 receptor 1:200, P2Y11 receptor 1:200, P2Y13 receptor 1:200, β-actin 1:1000). Blots were washed with TBS-T, incubated with secondary antibody (1:20,000) for 30 min at room temperature, and washed again with TBS-T. Specific proteins were visualized using ECL Western blotting detection reagents (GE Healthcare, Buckinghamshire, UK).

### Mobilization of intracellular calcium

Cells were washed twice, and then loaded with the Ca^2+^-sensitive fluorescent dye Fluo-4AM (Dojindo) in Ca^2+^-free HBSS for 30 min at 37°C. Cells were washed twice with Ca^2+^-free HBSS and exposed to UVA. Fluo-4 fluorescence was analyzed by using a Fluoroskan Ascent microplate fluorometer (Thermo scientific, Waltham, MA; excitation/emission 485 nm/527 nm).

### Statistics

Results are expressed as mean ± SD. The statistical significance of differences between two groups was calculated by using the unpaired Student's t-test, and multiple groups were compared using ANOVA followed by pairwise comparisons with Bonferroni’s *post hoc* analysis. Calculation was done with GraphPad prism 6.0 (GraphPad Software, San Diego, CA). The criterion of significance was set as P < 0.05.

## Results

### UVA-induced increases in IL-6 mRNA and protein levels in HaCaT cells

**Fig 1:**
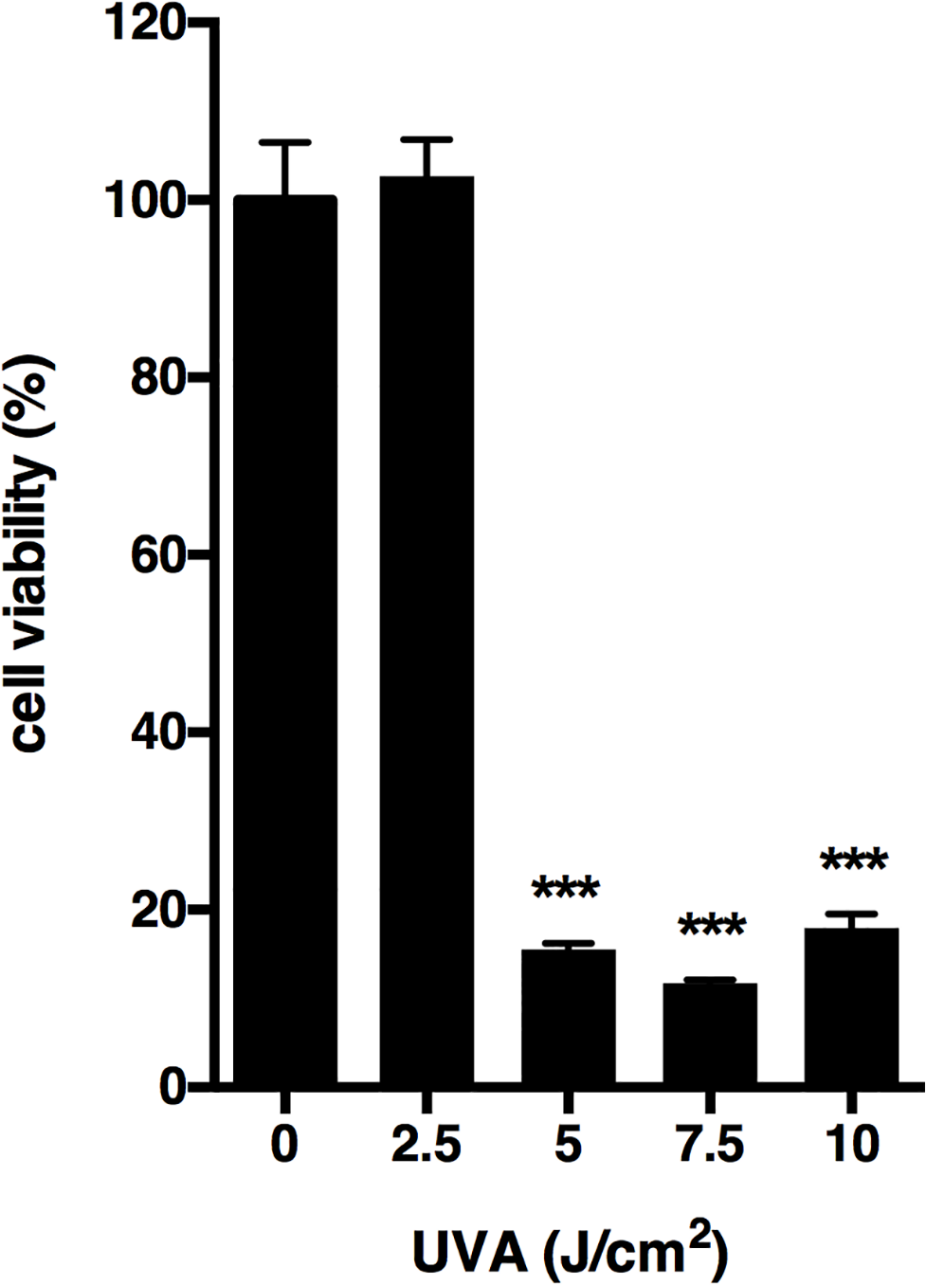
Effect of UVA irradiation on cell viability in HaCaT cells. Cells were irradiated with 2.5–10 J/cm^2^ UVA and incubated for 24 h. At the end of incubation, cell viability was determined by MTS assay as described in Materials and Methods. Each value represents the mean ± SD (n = 6). Significant differences between the irradiated groups and the non-irradiated groups are indicated by *** (P<0.001).

First, the effect of UVA irradiation on the viability of HaCaT cells was examined using MTS assay. Cells were irradiated with various doses of UVA (2.5, 5, 7.5, 10 J/cm^2^). The viability of cells irradiated with 5 J/cm^2^ UVA or more was significantly decreased, while the viability of cells irradiated with 2.5 J/cm^2^ UVA was not reduced (Fig 1).

**Fig 2:**
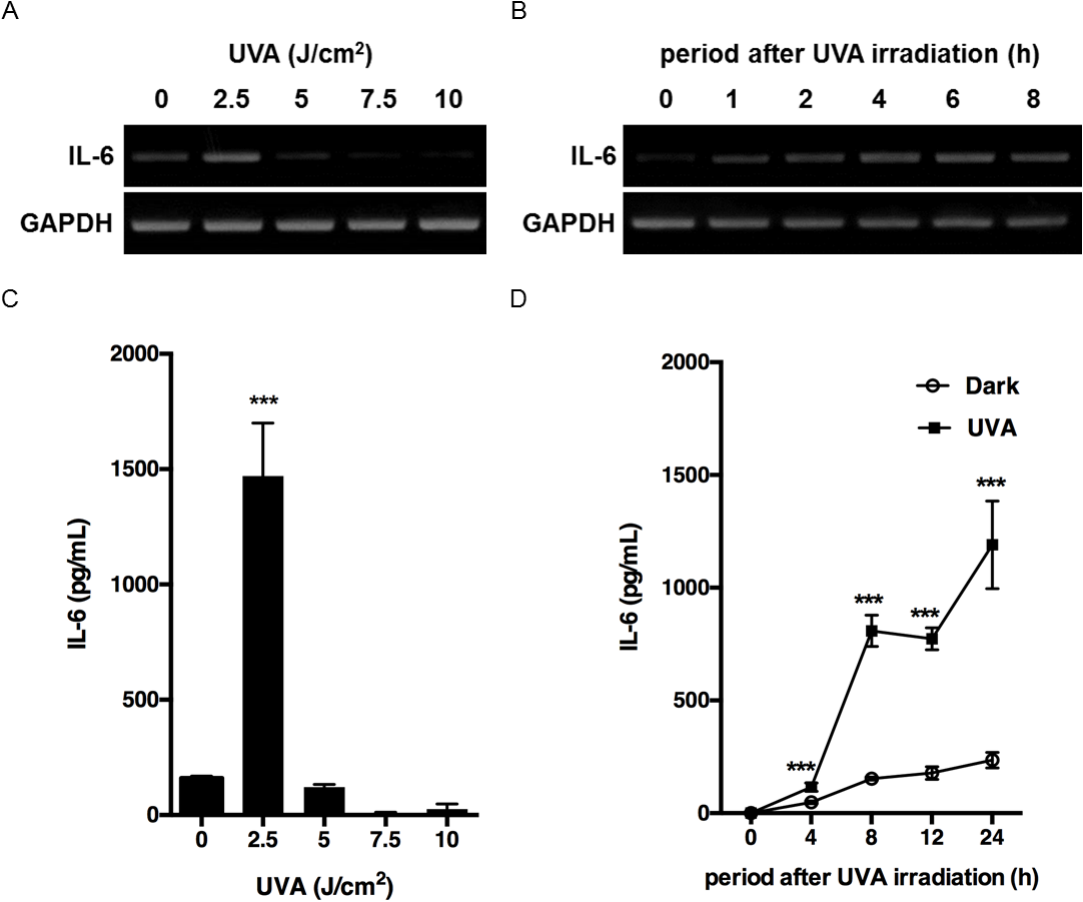
UVA-induced increase in IL-6 mRNA and protein levels in HaCaT cells. (A) Cells were irradiated with 2.5–10 J/cm^2^ and incubated for 4 h. (B) Cells were irradiated with 2.5 J/cm^2^ and incubated for the indicated times. In both experiments, IL-6 mRNA levels were analyzed by RT-PCR as described in Materials and Methods. (C) Cells were irradiated with 2.5–10 J/cm^2^ and incubated for 24 h. (D) Cells were irradiated with 2.5 J/cm^2^ and incubated for the indicated times. The culture supernatants were harvested and the concentration of IL-6 was measured as described in Materials and Methods. Each value represents the mean ± SD (n = 4). Significant differences between the irradiated groups and the non-irradiated groups are indicated by *** (P<0.001).

Next, we determined IL-6 production in response to UVA irradiation in HaCaT cells at the same dose levels. We found that 2.5 J/cm^2^ UVA increased IL-6 mRNA and IL-6 protein levels (Fig 2A and 2C), whereas higher UVA dosages did not, presumably because of their effect on cell viability. UVA at 2.5 J/cm^2^ also increased IL-6 mRNA and IL-6 protein levels in a time-dependent manner, with peaks at 6 and 24 h after irradiation, respectively (Fig 2B and 2D).

### Involvement of ATP released into extracellular space in UVA-induced IL-6 production

**Fig 3:**
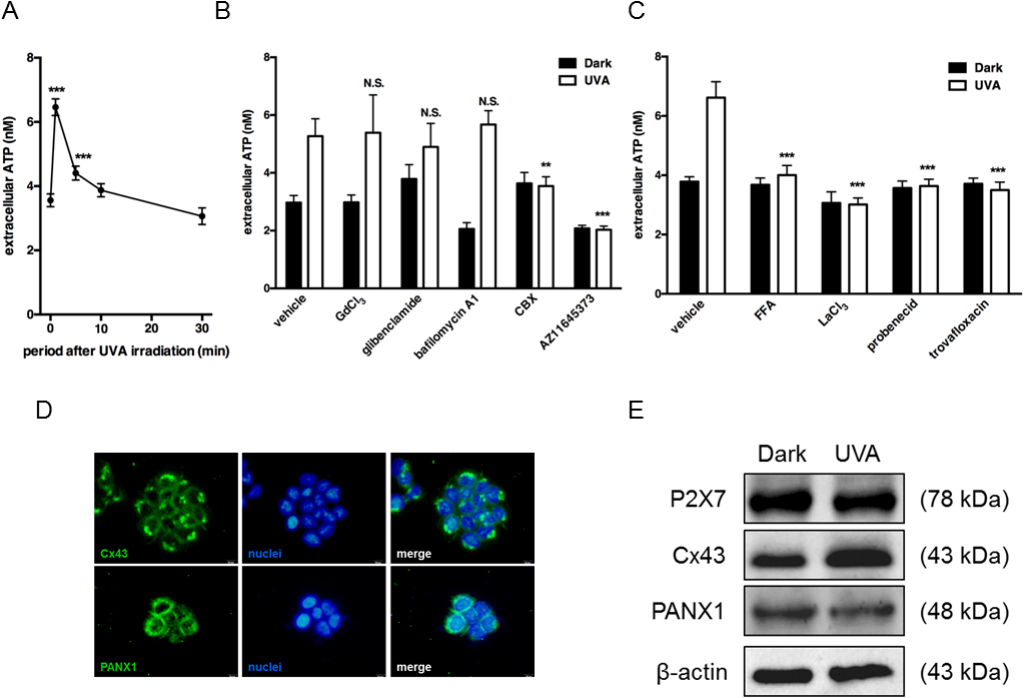
ATP release from UVA-irradiated HaCaT cells. (A) Cells were irradiated with 2.5 J/cm^2^ UVA and then incubated for the indicated times. (B) Cells were irradiated with 2.5 J/cm^2^ UVA after pretreatment with GdCl_3_ (50 μM), glibenclamide (100 μM), bafilomycin A1 (50 nM), CBX (20 μM), or AZ11645373 (10 μM) for 30 min, and culture supernatants were collected at 1 min after UVA irradiation. (C) Cells were irradiated with 2.5 J/cm^2^ UVA after pretreatment with FFA (50 μM), LaCl_3_ (200 μM), probenecid (500 μM), or trovafloxacin (30 μM) for 30 min, and culture supernatants were collected at 1 min after UVA irradiation. In each experiments, the concentration of ATP was measured as described in Materials and Methods; each value represents the mean ± SD (n = 8–12). Significant differences between the time point 0 or irradiated control groups and the indicated groups are indicated by ** (P<0.01) and *** (P<0.001). (D) Intracellular localization of Cx43 and PANX1 were detected by immunostaining (green, Cx43 or PANX1; blue, nuclei). (E) Cells were irradiated with 2.5 J/cm^2^ and incubated for 24 h. Protein expression of P2X7 receptor, Cx43 and PANX1 in non-irradiated and irradiated cells was compared by means of immunoblotting.

**Fig 4:**
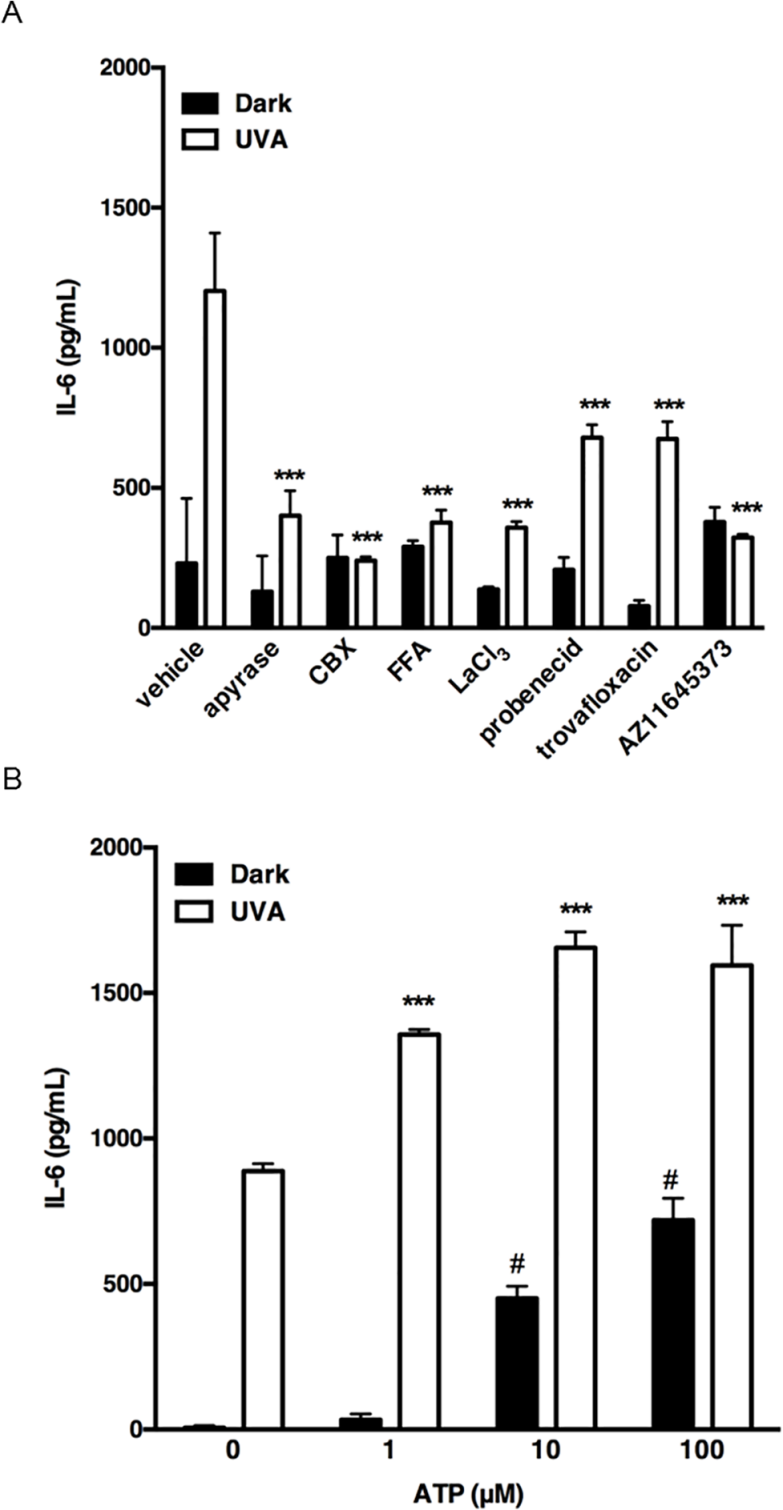
Involvement of released ATP in UVA-induced IL-6 production. (A) Cells were pre-incubated with apyrase (10 U/mL), CBX (20 μM), FFA (50 μM), probenecid (500 μM), LaCl_3_ (200 μM), trovafloxacin (30 μM) or AZ11645373 (10 μM) for 30 min, and irradiated with 2.5 J/cm^2^. (B) Cells were treated with 1–100 μM ATP at 1 min after UVA irradiation (2.5 J/cm^2^). After incubation for 24 h, the culture supernatants were harvested and the concentration of IL-6 was measured. Each value represents the mean ± SD (n = 4). Significant differences between the irradiated control groups and the indicated groups are indicated by *** (P<0.001). Significant differences between the control groups and the ATP-treated groups are indicated by # (P<0.001).

To examine UVA-induced release of ATP from HaCaT cells, we measured the concentration of ATP released from cells into culture medium after irradiation with UVA at 2.5 J/cm^2^. The extracellular concentration of ATP increased in response to irradiation, peaked just after irradiation, and then decreased to the basal level within 10 min (Fig 3A). To identify the pathway of the ATP release, we examined the effects of various inhibitors. GdCl_3_ (a maxi-anion channel blocker), glibenclamide (an anion transporter blocker), and bafilomycin A1 (an exocytosis blocker) had no effect on the ATP release. However, CBX (a nonspecific gap junction inhibitor) and AZ11645373 (a P2X7 receptor antagonist) significantly blocked the UVA-induced ATP release (Fig 3B). CBX blocks both connexin (e.g. Cx43) and pannexin (e.g. PANX1) hemichannels. Previous studies have provided evidences that both Cx43 and PANX1 could be involved in keratinocyte ATP release in response to other stimuli [18, 25]. To characterize the hemichannel subtypes involved in ATP release, we treated cells with FFA or LaCl_3_ (Cx43 inhibitors), or probenecid or trovafloxacin (PANX1 inhibitors). ATP release induced by UVA irradiation was significantly blocked by each of these inhibitors (Fig 3C). Immunofluorescence microscopy showed that Cx43 and PANX1 were expressed between adjacent cells to mediate cellular coupling, while some of them were exposed to the extracellular space (Fig 3D). In addition, the protein expression of P2X7 receptor, Cx43 and PANX1 in non-irradiated and irradiated cells were confirmed by immunoblotting. UVA irradiation enhanced Cx43 expression, but the expression level of P2X7 receptor and PANX1 was not affected by UVA (Fig 3E). These results indicate that Cx43 and PANX1channels, and P2X7 receptor mediate release of ATP from UVA-irradiated HaCaT cells. To examine the effect of inhibition of ATP release, we treated cells with CBX, FFA, LaCl_3_, probenecid, trovafloxacin or AZ11645373. The UVA-induced IL-6 production was abolished by each of these inhibitors (Fig 4A). The effect of ATP depletion on IL-6 production was also examined. Pretreatment with ecto-nucleotidase apyrase significantly suppressed IL-6 production, suggesting that endogenously released ATP mediates UVA-induced IL-6 production (Fig 4A). To further confirm the involvement of ATP in IL-6 production, we stimulated irradiated cells with ATP at 1 min after UVA irradiation. Fig 4B showed that low concentration of ATP (1 μM) alone didn’t trigger IL-6 production, but UVA-induced IL-6 production was significantly increased by post-treatment with 1 μM ATP. These results imply that release of ATP via Cx43 and PANX1channels, and P2X7 receptor might play an important role in UVA-radiation-induced IL-6 production in HaCaT cells.

### Effect of P2 receptor antagonists on UVA-induced IL-6 production

**Fig 5:**
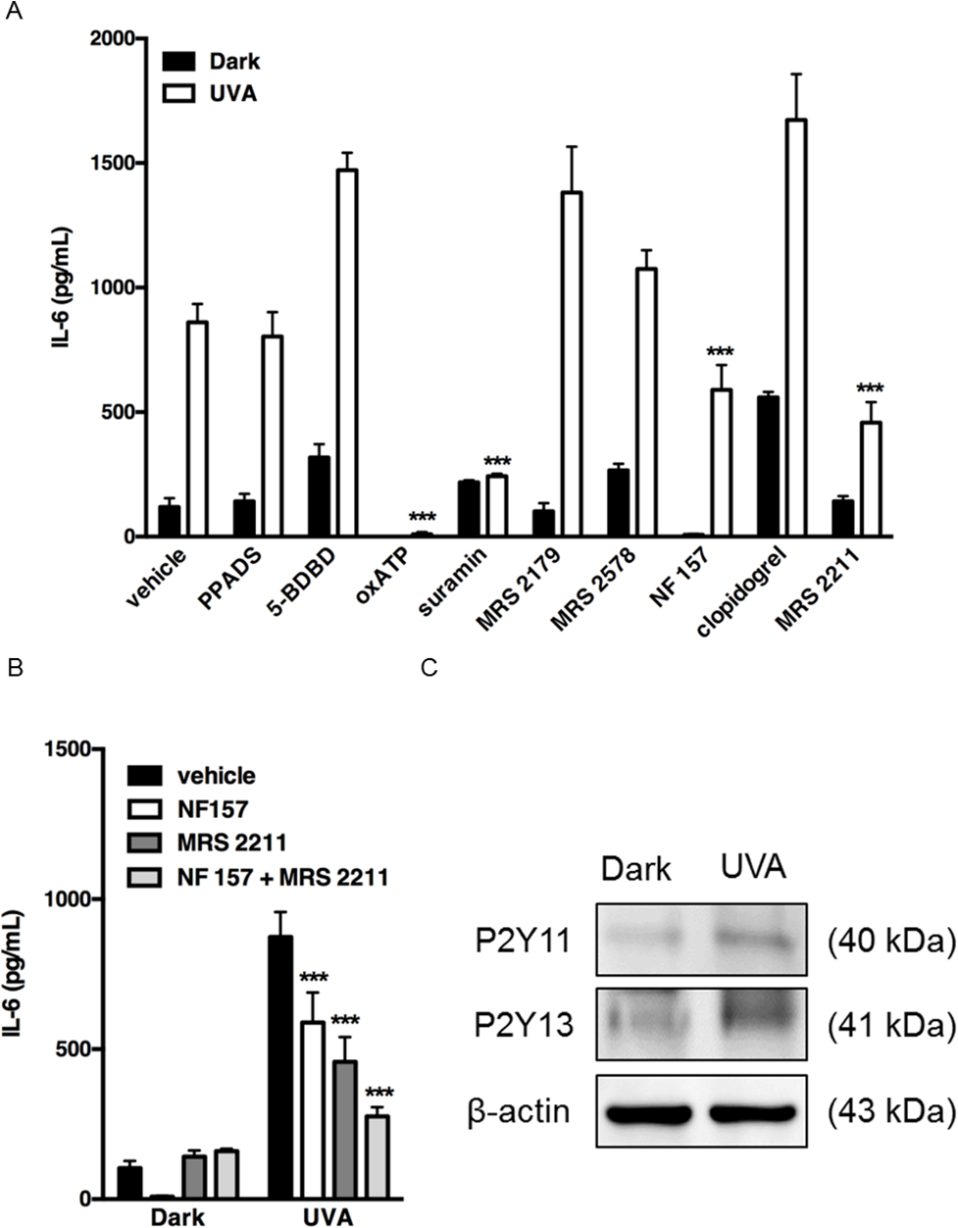
Involvement of P2 receptors in UVA-induced IL-6 production. Effects of P2 receptor antagonists on IL-6 production. (A) Cells were pre-incubated with PPADS (100 μM), 5-BDBD (2 μM), oxATP (100 μM), suramin (100 μM), MRS2179 (100 μM), MRS2578 (100 μM), NF157 (50 μM), clopidogrel (30 μM) or MRS 2211 (100 μM) for 30 min, and irradiated with 2.5 J/cm^2^. (B) Cells were pre-incubated with NF157 (50 μM) and/or MRS 2211 (100 μM) for 30 min, and irradiated with 2.5 J/cm^2^. In each experiment, the culture supernatants were harvested after incubation for 24 h and the concentration of IL-6 was measured. Each value represents the mean ± SD (n = 8–16). Significant differences between the irradiated control groups and the indicated groups are indicated by *** (P<0.001). (C) Cells were irradiated with 2.5 J/cm^2^ and incubated for 24 h. Protein expression of P2Y11 and P2Y13 receptors in non-irradiated cells and irradiated cells was compared by means of immunoblotting.

HaCaT cells express various P2 receptors. To identify which of them might be responsible for UVA-radiation-induced IL-6 production, the effects of various P2 receptor antagonists on IL-6 production were examined. PPADS (broad-spectrum P2 receptor antagonist), 5-BDBD (P2X4 receptor antagonist), MRS2179 (selective P2Y1 receptor antagonist), MRS2578 (selective P2Y6 receptor antagonist) or clopidogrel (selective P2Y12 receptor antagonist) did not suppress IL-6 production. On the other hand, pretreatment with oxATP (irreversible antagonist of P2X7 receptor), suramin (broad-spectrum P2Y receptor antagonist), NF157 (selective P2Y11 receptor antagonist) or MRS2211 (competitive P2Y13 receptor antagonist) significantly decreased IL-6 production (Fig 5A). Co-treatment with NF157 and MRS2211 suppressed IL-6 production more potently than treatment with either inhibitor alone (Fig 5B). These results suggest that P2X7, P2Y11 and P2Y13 receptors all contribute to UVA-induced IL-6 production. We confirmed that P2Y11 and P2Y13 receptors were constitutively expressed, and UVA irradiation enhanced P2Y11 and Y13 receptor expression at the protein level (Fig 5C).

### Downstream mechanism of P2Y11 receptor activation in UVA-induced IL-6 production

**Fig 6:**
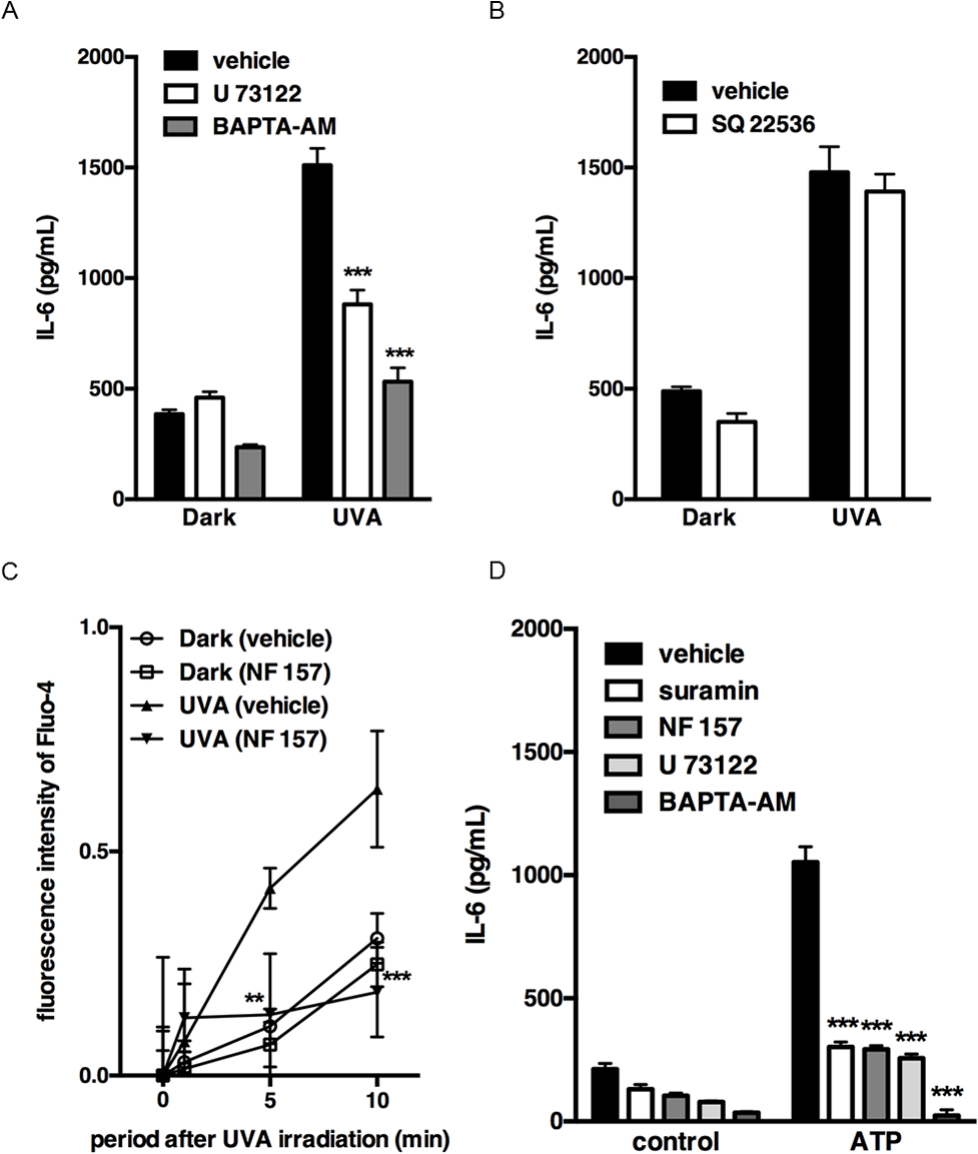
Involvement of elevation of intracellular Ca^2+^ in UVA-induced IL-6 production. Cells were pre-incubated with (A) U73122 (10 μM) or BAPTA-AM (50 μM) or (B) SQ22536 (10 μM), and irradiated with 2.5 J/cm^2^. After incubation for 24 h, the culture supernatants were harvested and the concentration of IL-6 was measured. Each value represents the mean ± SD (n = 4). (C) Cells loaded with Fluo-4 were irradiated with 2.5 J/cm^2^ in the absence or presence of NF 157 (50 μM). The fluorescence was analyzed with a fluorometer at the indicated times. Each value represents the mean ± SD (n = 6). (D) Cells were pre-incubated with suramin (100 μM), NF 157 (50 μM), U73122 (10 μM) or BAPTA-AM (50 μM), and stimulated with ATP (100 μM). After incubation for 24 h, the culture supernatants were harvested and the concentration of IL-6 was measured. Each value represents the mean ± SD (n = 4). Significant differences between the irradiated or ATP-treated control groups and the indicated groups are indicated by ** (P<0.01) and *** (P<0.001).

In order to analyze the signaling cascade upstream of IL-6 production, we focused on the pathway after P2Y11 receptor activation. P2Y11 receptor is linked to both G_q/11_ protein and G_s_ protein. To investigate the involvement of these proteins, we focused on elevation of intracellular Ca^2+^ following activation of phospholipase C (PLC) via G_q/11_ protein, and adenylate cyclase activation via G_s_ protein. U73122 (a PLC inhibitor) or BAPTA-AM (an intracellular Ca^2+^ chelator) significantly suppressed UVA-induced IL-6 production (Fig 6A), while SQ22536 (an adenylate cyclase inhibitor) had no effect (Fig 6B). These results suggested that the G_q/11_-PLC-Ca^2+^ pathway might be relevant to UVA-induced IL-6 production. We next examined whether UVA irradiation induces an increase of intracellular Ca^2+^ level. Elevation of intracellular Ca^2+^ was observed after irradiation, and this UVA-mediated effect was abolished by NF 157 (Fig 6C). We also examined the effect of treatment with P2Y11 receptor agonist ATP on IL-6 production. As shown in Fig 6D, ATP increased IL-6 production, and the ATP-induced IL-6 production was suppressed by pretreatment with NF157, U73122 or BAPTA-AM. These results suggested that UVA irradiation activates P2Y11 receptor, leading to IL-6 production through PLC-Ca^2+^-dependent signaling.

### Downstream mechanism of P2Y13 receptor activation in UVA-induced IL-6 production

**Fig 7:**
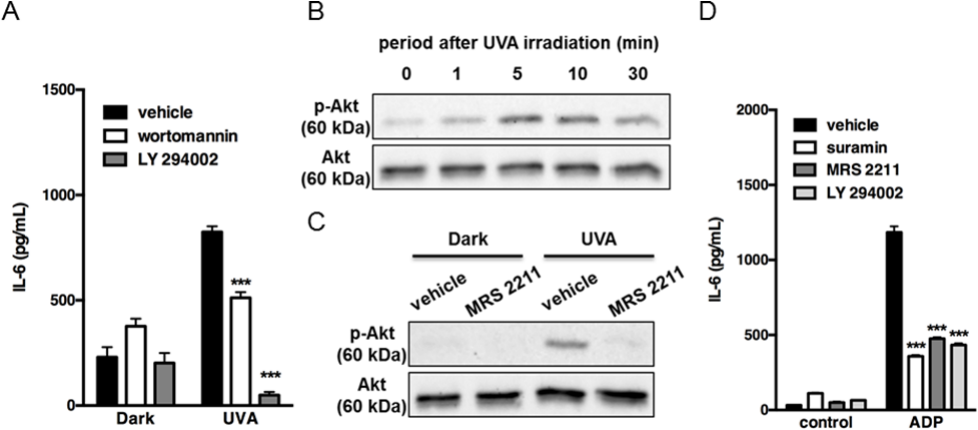
Involvement of Akt activation in UVA-induced IL-6 production. (A) Cells were pre-incubated with wortmannin (500 nM) or LY294002 (5 μM) for 30 min, and irradiated with 2.5 J/cm^2^. After incubation for 24 h, the culture supernatants were harvested and the concentration of IL-6 was measured. Each value represents the mean ± SD (n = 4). (B, C) Cells were irradiated with 2.5 J/cm^2^ and incubated for the indicated times (B). Cells were pre-incubated with MRS2211 (100 μM) for 30 min, and irradiated with 2.5 J/cm^2^, and then proteins were extracted after incubation for 10 min (C). In each experiment, phosphorylation of Akt was detected by immunoblotting as described in Materials and Methods. Equal protein loading was confirmed using anti-Akt antibody. The results are typical of those obtained in three independent experiments. (D) Cells were pre-incubated with suramin (100 μM), MRS2211 (100 μM) or LY294002 (5 μM) for 30 min, and stimulated with ADP (100 μM). After incubation for 24 h, the culture supernatants were harvested and the concentration of IL-6 was measured. Each value represents the means ± SD (n = 4). Significant differences between the irradiated or ADP-treated control groups and the indicated groups are indicated by *** (P<0.001).

In parallel experiments, the signaling pathway linked to P2Y13 receptor was studied. P2Y13 receptor is a Gi-coupled receptor with high affinity for ADP [26]. It has been reported that Gi signaling pathways are required for activation of Akt in platelets [27]. Therefore, we investigated the contribution of the PI3K/Akt pathway in UVA-induced IL-6 production by using a specific inhibitor, wortmannin, and LY294002. Both treatments significantly suppressed IL-6 production in response to UVA irradiation (Fig 7A). Next, the effect of UVA irradiation on Akt phosphorylation was examined. Phosphorylation of Akt was observed at 5 min after UVA irradiation (Fig 7B) and was suppressed by pretreatment with MRS2211 (Fig 7C). Similar effects were observed when P2Y13 receptor agonist ADP was applied. As shown in Fig 7D, ADP significantly facilitated IL-6 production and this effect was suppressed by pretreatment with MRS2211 or LY294002. These results suggested that UVA irradiation triggers IL-6 production through the P2Y13 receptor-PI3K/Akt-dependent pathway.

## Discussion

Although we have demonstrated the involvement of nucleotides in UVB-induced inflammatory signaling and activation of P2Y6 receptor [24], it is not known whether purinergic signaling contributes to UVA-radiation-induced responses. Our present results show that UVA irradiation of HaCaT cells induced ATP release. Since cell viability was unaffected by the same dose of UVA, cytosolic ATP must be released through a non-lytic mechanism(s). Various pathways could be involved, and so we examined the effects of selective inhibitors. Treatment with gap junction inhibitors or P2X7 receptor antagonist decreased UVA-elicited ATP release, indicating that UVA irradiation induces release of ATP through gap junction hemichannels and P2X7 receptor. P2X7 receptor has unique characteristics. First, while other P2 receptors are activated by nucleotides at micromolar order of concentration, P2X7 receptor activation requires millimolar levels of extracellular ATP [28]. Second, P2X7 receptor participates in not only intracellular inflammatory signaling, but also ATP release because of its interaction with hemichannels [29]. A recent study showed that ATP release via Cx43 and PANX1, widely expressed gap junction hemichannels in many cell types, occurred downstream of P2X7 receptor [30, 31]. Considering previous reports and the present data, we consider that P2X7 receptor participates in IL-6 production via induction of ATP release, rather than via a contribution to intracellular signaling. Our results indicated that both Cx43 and PANX1 are constitutively expressed, and mediate ATP release from UVA-irradiated HaCaT cells. The expression of Cx43 appeared to be modulated by UVA [32], although there has been no report of the influence of UVA on PANX1 function. Many other hemichannel subtypes (e.g. Cx26 and 31) are present in keratinocytes [33] and may also contribute to ATP release. More detailed analyses would be necessary to determine which specific connexin and pannexin channel subtypes are involved.

Extracellular nucleotides induce IL-6 production in human keratinocytes [21, 22]. Therefore, to investigate the role of nucleotides released from cells in IL-6 production in response to UVA irradiation, we examined the effect of apyrase, an ecto-nucleotidase. The increase of IL-6 production was significantly inhibited by apyrase, suggesting that nucleotides released from cells after UVA irradiation trigger IL-6 production. This notion was supported by the finding that post-treatment with ATP facilitated IL-6 production. Activation of P2 receptors requires a micromolar concentration range of nucleotides. Since nucleotides released from cells would be diluted in the culture supernatant and would also be rapidly metabolized by ecto-nucleotidases on the plasma membrane, the ATP concentration at the cell surface after UVA irradiation is likely to be substantially higher than that detected here. Therefore, we speculate that extracellular nucleotides would have been released in sufficient quantity to activate P2 receptors on UVA-irradiated HaCaT cells. Indeed, our results clearly showed that P2Y11 and P2Y13 receptors participate in UVA-induced IL-6 production. IL-6 production from UVA-irradiated cells was not completely blocked in the presence of ecto-nucleotidase, ATP release-inhibitors or P2Y antagonists. Although nucleotide-independent IL-6 production mechanisms were also conceivable, this is the first observation that purinergic signaling has an important role in UVA-mediated IL-6 production.

Various events upstream of IL-6 production have been reported. P2Y11 receptor is coupled to G_q/11_ protein, promoting PLC activation and subsequent mobilization of intracellular Ca^2+^. Sustained increase of intracellular Ca^2+^ might serve as a regulatory factor of various signaling pathways. Although it has been shown that UVA radiation mobilizes Ca^2+^ in HaCaT cells [34], it had not been established whether an increase of Ca^2+^ is involved in UVA-induced IL-6 production. Here, we showed that the increase of Ca^2+^ after UVA exposure was blocked by NF157, and mobilization of intracellular Ca^2+^ was required for UVA-induced IL-6 production. We concluded that UVA irradiation is likely to activate G_q/11_-coupled P2Y11 receptor to mobilize intracellular Ca^2+^ after PLC stimulation. On the other hand, P2Y13 receptor is a G_i_ protein-coupled receptor. Activation of G_i_ may stimulate distinct intracellular signals, i.e., the mitogenic Ras-MAPK cascade and the PI3K/Akt pathway [35, 36]. Overall, our results show that UVA-induced IL-6 production is dependent on the P2Y13 receptor-PI3K/Akt signaling pathway. Previous studies have indicated that PI3K/Akt is a crucial event in P2Y13 signaling [37], and that G_i_-mediated PI3K activation phosphorylates downstream Akt and subsequently induces IL-6 production in ovarian cancer cells [38]; these results strongly support our conclusions. It has also been shown that p38 MAPK is an important intracellular mediator of UVA-induced IL-6 production [39]. Our data indicate that p38 MAPK is independent of activation of P2Y13 receptor, since MRS2211 did not suppress phosphorylation of MAPKAPK-2, which is downstream of p38 MAPK (S1 Fig). P2Y13 receptor has been also reported to mediate Ca^2+^ signaling [40]. However, since MRS2211 did not affect UVA-induced intracellular Ca^2+^ response (S2 Fig), we concluded that P2Y13-Akt signaling and Ca^2+^ response are independent phenomena. The PI3K/Akt signaling pathway regulates the activity of the transcriptional factor nuclear factor-kappa B (NF-κB), and NF-κB is targeted by Akt in anti-apoptotic signaling and carcinogenesis [41]. Thus, inhibition of PI3K/Akt signaling by P2Y13 receptor antagonist may have role in inhibition of UVA-induced skin tumor growth.

UV-induced IL-6 expression increases keratinocytes proliferation and skin inflammation [42, 43]. IL-6 can also reinforce the senescent growth arrest [44] and induce MMPs via an autocrine mechanism [15]. UV-induced MMP-1 expression increases extracellular matrix protein degradation, resulting in photoaging [8]. Indeed, the basal level of IL-6 and MMP-1 in photoaged skin is higher than in normal skin [45, 46]. Further study will be needed to determine whether purinergic signaling is responsible for the expression of photoaging markers such as MMP-1.

In conclusion, we found that both P2Y11 and P2Y13 receptors were coupled to UVA-induced IL-6 production, activating different intracellular signaling cascades that involve Ca^2+^ for P2Y11 and PI3K/Akt for P2Y13. P2X7 receptor is also involved in IL-6 production via mediation of ATP release in response to UVA irradiation. These data indicate that nucleotides could contribute to skin inflammation via autocrine/paracrine signaling after UVA irradiation. This in turn raises the possibility that inhibition of purinergic signaling may be a novel strategy to prevent or ameliorate skin damage in response to UVA radiation.

## Supporting Information

S1 Fig P2Y13 receptor antagonist MRS2211 did not suppress UVA-induced phosphorylation of MAPAPK-2. Cells were irradiated with 2.5 J/cm^2^ and incubated for the indicated times (Figure A). Cells were pre-incubated with MRS2211 (100 μM) for 30 min, and irradiated with 2.5 J/cm^2^. After incubation for 60 min, proteins were extracted (Figure B). Phosphorylation of p38 MAPK and MAPAPK-2 was detected by immunoblotting. Equal protein loading was confirmed using anti-p38 MAPK and MAPKAPK-2 antibody. The results are typical of those obtained in three independent experiments.
(TIF)

S2 Fig P2Y13 receptor antagonist MRS2211 did not suppress UVA-induced intracellular Ca^2+^ response. Cells loaded with Fluo-4 were irradiated with 2.5 J/cm^2^ in the absence or presence of MRS2211 (100 μM). The fluorescence was analyzed with a fluorometer at the indicated times. Each value represents the mean ± SD (n = 6).
(TIFF)
