# Supplementary figures and images for: Autocrine Regulation of UVA-Induced IL-6 Production via Release of ATP and Activation of P2Y Receptors
Source: PLoS One. 2015 Jun 1;10(6):e0127919. doi: 10.1371/journal.pone.0127919 (PMC4452185; doi:10.1371/journal.pone.0127919)

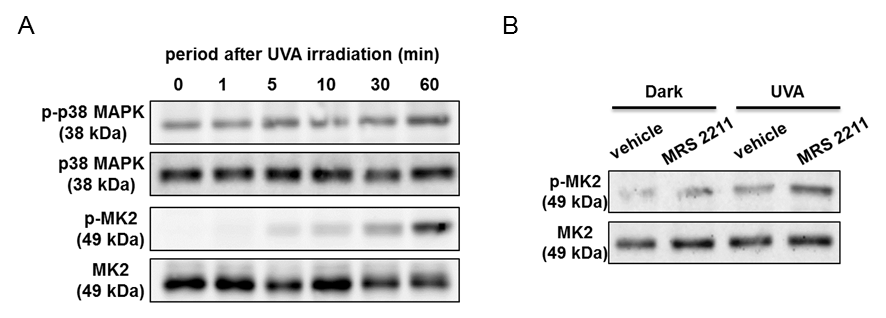

Supplement: S1 Fig — Cells were irradiated with 2.5 J/cm2 and incubated for the indicated times (Figure A). Cells were pre-incubated with MRS2211 (100 μM) for 30 min, and irradiated with 2.5 J/cm2. After incubation for 60 min, proteins were extracted (Figure B). Phosphorylation of p38 MAPK and MAPAPK-2 was detected by immunoblotting. Equal protein loading was confirmed using anti-p38 MAPK and MAPKAPK-2 antibody. The results are typical of those obtained in three independent experiments. (TIF) [file pone.0127919.s001.tif]

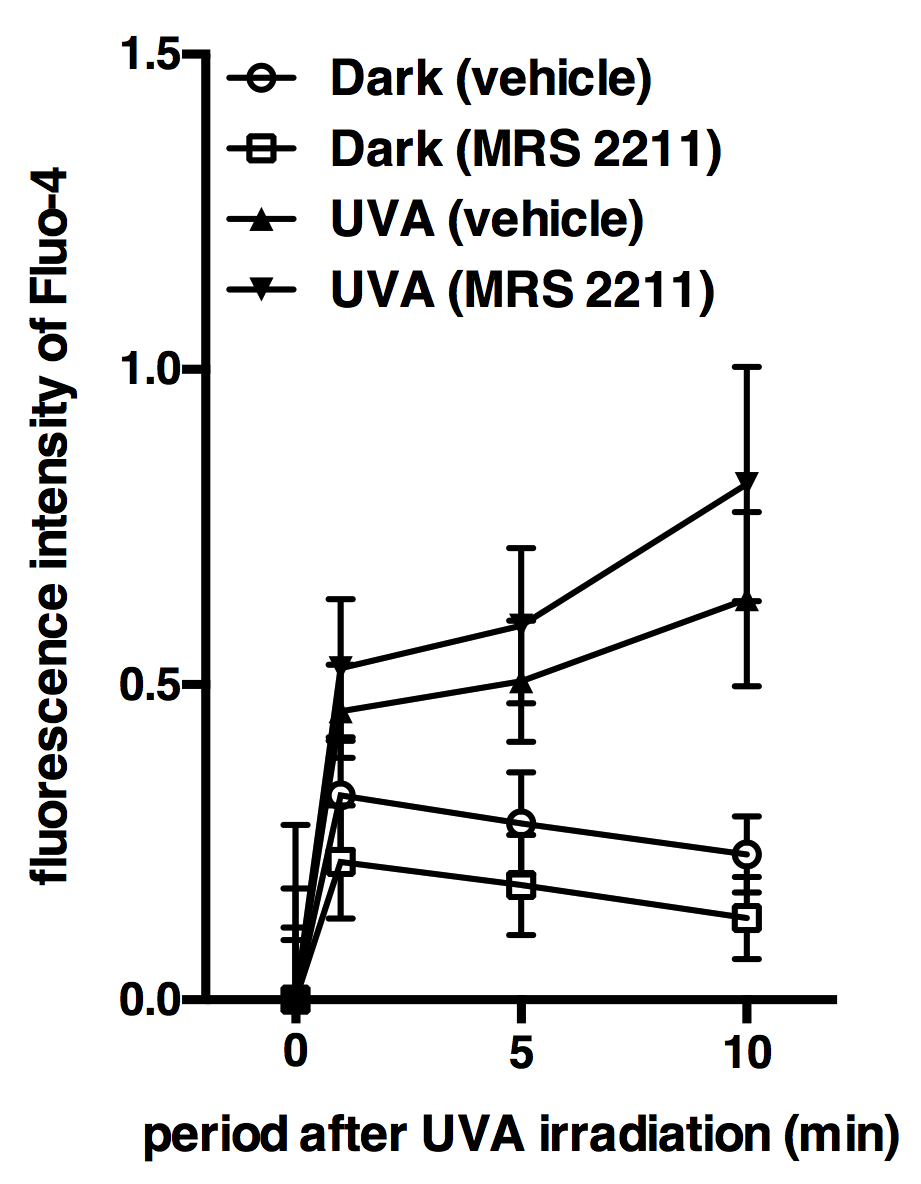

Supplement: S2 Fig — Cells loaded with Fluo-4 were irradiated with 2.5 J/cm2 in the absence or presence of MRS2211 (100 μM). The fluorescence was analyzed with a fluorometer at the indicated times. Each value represents the mean ± SD (n = 6). (TIFF) [file pone.0127919.s002.tiff]
